# Histopathological features predict metastatic potential in locally advanced colon carcinomas

**DOI:** 10.1186/s12885-015-1013-7

**Published:** 2015-01-21

**Authors:** Caren Jayasinghe, Nektaria Simiantonaki, Charles James Kirkpatrick

**Affiliations:** 1 Institute of Pathology, Johannes Gutenberg University, Langenbeckstr. 1, 55101 Mainz, Germany; 2 Department of Pathology, Laboratory Medicine Cologne, Geibelstr. 2, 50931 Cologne, Germany; 3 Institute of Pathology Essen-Mitte, Am Deimelsberg 34a, 45276 Essen, Germany

**Keywords:** Tumor budding, Inflammation, Desmoplasia, Necrosis, Blood vessel density, Lymphatics, Colon cancer, Metastasis

## Abstract

**Background:**

Metastatic dissemination can exist before a pathologically and clinically detectable manifestation. The structural heterogeneity of colon cancer (CC) in histological sections with respect to the morphology of tumor aggressiveness and composition of the tumor microenvironment raises the question of whether the microscopical tumor architecture enables a discrimination of groups with different metastatic potential. This would result in an assessment of the prognosis and provision of an ancillary tool for the therapeutic management after surgery, beside the estimation of the local tumor extent.

**Methods:**

In order to identify predictive biomarkers for metastasis of locally advanced CC, which can easily be integrated into the pathologist’s daily routine diagnostic activity, we determined tumor budding, peritumoral inflammation, extent of desmoplasia and necrosis, density of macro- and microvascular blood vessels and functional state of lymphatics in the tumor center, invasive margin and tumor-free surrounding tissue in 86 non-metastatic, lymphogenous-metastatic and haematogenous-metastatic, subserosa-invasive CC.

**Results:**

Features influencing nodal metastasis in the univariate analysis included high tumor budding (p = 0.004), high large vessel density in the subserosa (p = 0.043), abundant desmoplasia (p = 0.049), non-finger-like desmoplastic pattern (p = 0.051) and absent lymphocellular intratumoral inflammation (p = 0.084). In the multivariate analysis, with the exception of large vessel density, these pathomorphological features were independent risk factors for lymphogenous metastasis (p = 0.023, p = 0.017, p = 0.037, p = 0.012, respectively) with a good discrimination ability (AUC of 0.853). Features associated with distant metastasis in the univariate analysis included high tumor budding (p = 0.002), low intratumoral small vessel density (p = 0.013), absent lymphocellular intratumoral inflammation (p = 0.048) and abundant necrosis (p = 0.073). In the multivariate analysis only tumor budding was an independent predictor for haematogenous metastasis (p = 0.007) with a good discrimination ability (AUC of 0.829).

**Conclusions:**

Thus, mainly tumor budding but also the described structural characteristics of the peritumoral tissue appears to reflect the metastatic potential of locally advanced CC and therefore should be stated in pathological reports.

## Background

The metastatic state of colon carcinoma (CC) determines its prognosis. The 5-year survival rate of patients with distant metastasis is only about 10% compared to approximately 60% with lymphogenous metastasis and over 80% without metastasis [1]. Notably, metastatic dissemination could exist before a pathologically and clinically detectable manifestation. Hence the question arises, whether a detailed analysis of the microscopical tumor architecture on conventional histological sections and the recognition of potential structural differences between non-metastatic and metastatic cases could contribute to determine the metastatic potential of CC, beside the local tumor extent. Indeed, in histological sections a structural heterogeneity of CC can be seen within the different zones of the same tumor and between tumors of the same infiltrating depth and grading.

The main histopathological characteristics of carcinoma include the invasive growth pattern and the associated changes in the surrounding stroma such as stromal desmoplasia, peritumoral inflammatory reaction and necrosis. These structural features reflect the interaction between tumor cells and the “tumor microenvironment”, which may promote tumor growth and progression [2,3].

CC show different growth patterns at the invasive front. This so-called tumor budding is predictive of metastasis, local recurrence and survival in CC [4]. As an adverse prognostic factor tumor budding can help to stratify patients into more meaningful risk groups than TNM staging alone, as well as for adjuvant chemotherapy in low tumor stages [5]. Despite these data, a statement in the histopathological report concerning the degree of tumor budding has not yet been widely applied.

During cancer growth, tumors not only destroy the pre-existing extracellular matrix, but usually actively create their own stroma, the so-called desmoplastic stroma [6]. Desmoplasia may represent a barrier against cancer diffusion or a stroma to build up and support the tumor. Its role is controversially discussed regarding a tumor-protecting versus host-protecting impact.

The presence of immuno-inflammatory cells in CC is a common phenomenon [7]. The inflammatory response can have dual effects in the progression of cancer. On one side inflammation can promote tumor progression by various pro-inflammatory cytokines that activate tumorigenic pathways leading to increased growth and survival of tumor cells [8]. On the other hand, inflammatory cells have a host-protecting function in the sense of anti-tumor immunity. In CC lymphocytic reactions are independent prognostic factors for a better survival [9,10]. However, whether and which immune infiltrates have an effect on the metastatic potential of CC is not clear.

CC frequently shows characteristic necrotic debris in glandular lumina, so-called “dirty necrosis”. Tumor necrosis is seen in rapidly proliferating tumors outgrowing their blood supply, becoming hypoxic and finally necrotic. In turn, release of growth-, survival- and angiogenic factors support tumor progression and metastasis [11]. Tumor necrosis has been proposed as a marker of poor prognosis in carcinomas of various histogenetical origins [12]. In CC, first histological studies provided evidence of tumor necrosis as a parameter associated with progressive tumor behavior [13,14].

Tumor vasculature, the network of blood and lymphatic vessels in and around a growing neoplasm is a basic component of solid tumors is the tumor vasculature. The vessel system is essential for adequate tumor tissue oxygenation and nutritional supply but also for metastatic spread [15,16]. Despite the fact that vascular anatomy in tumor tissue represents an important subject for tumor growth and metastasis, reports with a detailed histoarchitectural analysis in human tissue are rare.

In order to determine characteristic structural features that could be associated with the process of metastasis we investigated the histomorphology of the tumor and its microenvironment in 86 non-metastatic, lymphogenous and haematogenous metastatic, moderately differentiated, subserosa-invasive CC. We characterized the grade of tumor budding, the cell types of the intra- and peritumoral inflammation as well as the extent and pattern of desmoplasia and necrosis. An essential part of our work was the analysis of the density of intra- and peritumoral as well as surrounding macro- and microvascular blood vessels. Additionally, we determined the functional state of the tumor-associated lymphatic vessels. The major aim of our study was to identify relevant predictive biomarkers for the metastatic potential of CC on the basis of conventional histology and routinely applicable immunohistochemistry, which can easily be integrated into the pathologist’s report in addition to the TNM-classification. We show that conventionally treated primary tumor slides contain valuable ancillary information on tumor behavior apart from features such as tumor infiltration depth.

## Methods

### Ethics statement

For the investigation of CC tissues ethical approval was granted by the Clinical Research Ethics Committee of the federal state of Rhineland-Palatinate (Mainz, Germany). Written informed consent of all patients was obtained.

### Tissue samples

The colon tissue samples used in this study came from 86 patients with an average age of 65.2 (range 52–83) undergoing elective surgery for sporadic CC at the University of Mainz during the years 1998–2003. None of the patients received chemotherapy or radiation therapy before surgery. The morphological classification of the carcinomas was conducted according to WHO specifications. All tumors were staged following the guidelines of the TNM Classification of Malignant Tumors [17]. With respect to the T status all tumors investigated were T3 (infiltration of subserosa) and moderately differentiated (G2). They were separated into three groups according to metastatic status. The first group comprised 37 cases without tumor metastasis to regional lymph nodes or distant organs (N0/M0). Among the remaining metastasizing cases, 24 were characterized by lymphogenous (N+) and 25 by lymphogenous and haematogenous metastases (M+). TNM-status confirms the tumor state at the time of pathological diagnosis. Thus, the metastatic cases had already metastasized. The metastasizing organ was in all M+ cases the liver.

### Immunohistochemistry

All immunohistochemical reactions were conducted with formalin-fixed and paraffin-embedded samples.

*CD31, D2-40*: After deparaffination heat-induced epitope retrieval was performed in Tris-EDTA buffer pH 9,0 for 20 min. using a vegetable steamer. Non-specific binding was blocked by Dako REAL™ Peroxidase-Blocking Solution (Dako, Hamburg, Germany) prior to incubation with the primary antibody. For the immunohistochemical staining procedure DAKO REAL™EnVision™Detection System, Peroxidase/DAB+, Rabbit/Mouse was utilized following the provider’s instructions. The primary antibodies mouse monoclonal CD31 (Dako, Hamburg, Germany) and mouse monoclonal D2-40 (Signet, Dedham, MA, USA) were applied at a dilution of 1:50 for 1 h at room temperature. Sections were counterstained with Mayer’s haematoxylin.

*sm-actin*: After deparaffination the primary antibody mouse monoclonal sm-actin (Zytomed Systems, Berlin, Germany) was added at a dilution of 1:800 for 45 minutes at room temperature. UltraVision Quanto Detection System HRP DAB (Thermo Scientific, Fremont, CA, USA) was used for the staining procedure following the provider’s instructions. Sections were counterstained with Mayer’s haematoxylin.

### Histopathological analysis

Haematoxylin and eosin (H.E.)-stained slides of the tumor specimen were evaluated by two authors (CJ, NS) independently without knowledge of the TNM-status.

*Tumor budding* was defined as single tumor cells and oligocellular tumor cell clusters (≤5 cells) along the tumor invasion front. It was graduated according to the scoring system proposed by Nakamura et al.,[18] as none – low (< 1/3 of invasion front) – moderate (> 1/3 < 2/3 of invasion front) or strong (> 2/3 of invasion front).

*Inflammation* was determined on the basis of the type composition of the detected inflammatory cells in H.E. stained tissue. Four types of inflammatory reactions were distinguished: pure lymphocytic, mixed (lymphocytic/neutrophilic), pure neutrophilic and histiocyte-rich inflammation.

For *tumor-desmoplasia-necrosis tissue distribution* viable tumor, desmoplastic stroma and necrosis were estimated as percentage of the entire tumor section. The total of the three pathological features was 100%. Additionally tumor/desmoplasia and tumor/necrosis ratios were calculated.

The *desmoplastic pattern* along the invasive front and surrounding subserosal adipose tissue was divided into a pushing, finger-like or net-like pattern. Necrotic areas included stromal necroses and intraluminal necrotic debris of the neoplastic glands.

*Large vessel density (LVD)*: Arteries with a thick muscle wall were considered as large vessels. Two areas were counted at a low optical power (x 4 objective, field area 23.75 mm^2^). The final LVD was the mean value.

*Small vessel density (SVD)*: Vessels with narrow lumina but well definable up to five smooth muscle layers were considered as small vessels. Five areas were counted at medium optical power (x 10 objective, field area 3.80 mm^2^). The final SVD was the mean value.

*Microvascular density (MVD)*: Vessels with clearly defined lumina or linear vessel shape were regarded as microvessels. After immunohistochemical CD31 staining three highly vascularized areas (hot spots) were chosen at low optical power. Microvessel counting was performed at higher magnification within the hot spots (x 20 objective, field area 0.95 mm^2^). The final MVD was the mean value of three appraised high power fields.

LVD, SVD and MVD were determined intratumorally and along the invasion front, LVD and SVD additionally in tumor surrounding non-neoplastic subserosal adipose tissue.

*Lymphatics* were defined as D2-40-positive muscle layer-free vessels. Lymphatic vessels were evaluated according to the morphology of their lumina in the investigated tumor zone (compressed, mixed (compressed/open) and open).

### Statistical analysis

Data analysis was performed using SAS statistical software. Differences between groups with respect to categorical variables were analyzed by the following methods: Chi^2^ test, Fisher’s exact test and logistic regression. P-values, odds ratios (OR) and their 95% confidence intervals (CI) were used in the interpretation. Differences between groups with respect to continuous non-normal distributed variables were analyzed by the Wilcoxon rank-sum test. All relevant variables being significant (p < 0.05) or showing a strong tendency towards significance (p < 0.1) in the univariate analysis were included in a multivariate logistic model and plotted as receiver operating characteristic (ROC) curves to illustrate the predicted relevance. Additionally, we determined the intra- and interobserver agreement for the estimation of the tumor-desmoplasia-necrosis distribution with a test of reproducibility by calculating the Spearman’s rank correlation coefficient (r) and with a test of reliability by evaluating the weighted kappa coefficient (κ) in 22 cases.

## Results

To elucidate the relevance of the histomorphological features, tumor budding, peritumoral inflammation, desmoplasia, tumor-associated necrosis as well as morphology and density of tumor-associated blood and lymphatic vessels for CC metastasis, we investigated non-metastatic (N0/M0), lymphogenous-metastatic (N+), and haematogenous-metastatic (M+) CC. In order to document the structural and functional heterogeneity of tumor compartments we subdivided the tumor tissue into three zones, tumor center (zone 1), invasive margin (zone 2) and tumor-free surrounding tissue (zone 3). The tumor center represents the tumor compartment with the main mass of tumor with the most nutritional and oxygen requirements. The tumor margin represents the invasive front site and is a dynamic tumor-host interface. The surrounding tumor-free vessel-bearing subserosal adipose tissue ensures an adequate nutritional and oxygen supply.

### Univariate analysis

#### Tumor budding

The graduation of tumor budding along the invasive front according to the scoring system proposed by Nakamura et al. [18] is a simple, reproducible method with high interobserver accordance. With the conventional H.E. staining disseminated single tumor cells and oligocellular tumor clusters (≤5 tumor cells) at the invasive margin were clearly identifiable. As shown in Table 1 the degree of this histopathological factor varied depending on metastatic status. The dichotomization between low (0 to 1/3 of the invasion front) versus high (>1/3 of the invasion front) tumor budding revealed that high tumor budding correlates with increased metastatic status. Whereas in only 10% of the non-metastazing cases high budding was observed, the metastatic carcinomas revealed high budding in about 50% of the cases. The results were highly significant between N0/M0 versus N+ (p = 0.0037) as well as N0/M0 versus M+ (p = 0.0022) (Table 2).

**Table 1:** **Percentage distribution of histopathological features in non-metastatic (N0/M0), lymphogenous-metastatic (N+) and haematogenous-metastatic (M+) colon carcinomas**

	N0/M0	N+	M+
**Tumor budding**			
none	24	4	0
weak	64	50	52
moderate	6	17	28
strong	6	29	20
**Inflammation zone 1**			
none	3	21	16
granulocytic	3	0	8
mixed	46	33	28
lymphocytic	48	46	48
**Inflammation zone 2**			
none	0	8	0
granulocytic	3	0	0
mixed	35	13	20
lymphocytic	59	79	68
xanthogranulomatous	3	0	12
**Distribution (total 100%)**			
Tumor	52	50	48
Desmoplasia	34	38	35
Necrosis	14	12	17
**Tumor/desmoplasia ratio**			
> median	59	33	48
≤ median	41	67	52
**Tumor/necrosis ratio**			
> median	59	50	32
≤ median	41	50	68
**Desmoplasia pattern**			
finger-like	46	21	36
pushing	27	46	20
net-like	27	33	44

**Table 2:** **Univariate logistic regression analysis showing associations of histopathological features in non-metastatic (N0/M0) versus lymphogenous-metastatic (N+) and haematogenous-metastatic (M+) CC**

	N0/M0 vs. N+		N0/M0 vs. M+	
	OR (95% C.I.)	p-value	OR (95% C.I.)	p-value
**Tumor budding** low vs. high	6.98 (1.88-25.92)	**0.004**	7.61 (2.07-27.97)	**0.002**
**Inflammation zone 1** lymphocytic vs. non-lymphocytic	0.22 (0.04-1.23)	**0.084**	0.18 (0.03-0.99)	**0.048**
**Inflammation zone 2** lymphocytic vs. non-lymphocytic	0.63 (0.08-4.83)	NS	0.42 (0.06-2.71)	NS
**Tumor** percentage distribution	0.99 (0.96-1.03)	NS	0.98 (0.94-1.02)	NS
**Desmoplasia** percentage distribution	1.02 (0.98-1.06)	NS	1.01 (0.97-1.04)	NS
**Necrosis** percentage distribution	0.98 (0.93-1.02)	NS	1.01 (0.97-1.05)	NS
**Tumor/desmoplasia ratio** (> median, ≤median)	0.34 (0.12-0.99)	**0.049**	0.63 (0.23-1.75)	NS
**Tumor/necrosis ratio** (> median, ≤median)	0.68 (0.24-1.92)	NS	0.38 (0.13-1.09)	**0.073**
**Desmoplasia pattern** finger-like vs. non-finger-like	0.31 (0.09-1.01)	**0.051**	0.66 (0.23-1.87)	NS
**Large vessel density zone 1** (> median, ≤median)	1.89 (0.63-5.62)	NS	2.07 (0.71-6.06)	NS
**Large vessel density zone 2** (> median, ≤median)	2.61 (0.89-7.69)	NS	0,92 (0.33-2.57)	NS
**Large vessel density zone 3** (> median, ≤median)	3.11 (1.03-9.32)	**0.043**	1.47 (0.53-4.09)	NS
**Small vessel density zone 1** (> median, ≤median)	0.79 (0.28-2.28)	NS	0.24 (0.07-0.74)	**0.013**
**Small vessel density zone 2** (> median, ≤median)	0.58 (0.20-1.65)	NS	0.87 (0.31-2.42)	NS
**Small vessel density zone 3** (> median, ≤median)	1.30 (0.46-3.64)	NS	1.20 (0.43-3.33)	NS
**Microvascular vessel density zone 1** (> median, ≤median)	0.89 (0.32-2.51)	NS	0.87 (0.31-2.42)	NS
**Microvascular vessel density zone 2** (> median, ≤median)	1.37 (0.49-3.84)	NS	0.52 (0.81-1.45)	NS
**Altered vessels zone 1** present vs. absent	0.83 (0.28-2.45)	NS	0.93 (0.33-2.67)	NS
**Altered vessels zone 2** present vs. absent	1.41 (0.48-4.18)	NS	1.12 (0.37-3.35)	NS
**Altered vessels zone 3** present vs. absent	0.93 (0.32-2.76)	NS	0.74 (0.24-2.21)	NS
**Lymphatic vessels zone 1** open vs. compressed	0.29 (0.03-3.32)	NS	0.59 (0.03-10.11)	NS
**Lymphatic vessels zone 2** open vs. compressed	1.59 (0.26-9.56)	NS	1.22 (0.27-5.52)	NS
**Lymphatic vessels zone 3** open vs. compressed	1.62 (0.26-10.08)	NS	1.77 (0.32-9.71)	NS

Relevant variables being significant (p < 0.05) or showing a strong tendency towards significance (p < 0.1).

OR: odds ratio, C.I.: confidence interval, NS: no significant.

#### Inflammation

Using the conventional H.E. staining four types of inflammatory reaction in CC were identified: (1) pure lymphocytic, (2) pure granulocytic, (3) mixed granulocytic/lymphocytic and (4) histiocyte-rich. Inflammation were seen intratumorally (zone 1) and along the invasive front (zone 2). Lack of intratumoral inflammation effectively did not exist in non-metastatic cases (Table 1). In contrast, approximately 20% of the metastatic cases showed no intratumoral inflammation. Neutrophil granulocytes as exclusive inflammatory cells were seen only in three cases. A histiocyte-rich pattern was found in four cases, but only at the invasion front. In barely 50% of the cases, a lymphocellular infiltration was found in the tumor center, independent of the metastatic status. In a markedly higher percentage, a dominant, lymphocytic infiltration along the invasive margin in all comparative groups was demonstrated, especially in nodal positive carcinomas (79% of the cases). In about 50% of the non-metastatic and in approximately 30% of the metastatic cases a mixed type inflammation was seen in the intratumoral region. This inflammation pattern was observed less commonly at the tumor margin.

Comparing lymphocyte-associated (lymphocytic/mixed type) inflammation versus none/pure granulocytic reaction in zone 1, the results were just below statistical significance in the nodal positive status (p = 0.084) but significant in the cases of distant metastasis (p = 0.048) (Table 2). The inflammation type in zone 2 showed no significant differences between non-metastasized and metastasized cases (Tables 1 and 2).

#### Necrosis and Desmoplasia

For each tumor specimen the percentage of viable tumor, desmoplastic stromal reaction and necrosis was estimated. The estimation of the percentage distribution tumor-desmoplasia-necrosis was performed with a semiquantitative scoring system. The reproducibility of the intra- and interobserver variation was excellent and good, respectively (r = 0.817 for intraobserver variation and r = 0.771 for interobserver variation; p < 0.001). Both, the reliabilities for the intraobserver variation [κ = 0.799 (95% C.I.:0.577-1)] and for interobserver variation [κ = 0.753 (95% C.I.:0.520-0.987)] were good. The distribution of these three histopathological features did not reveal significant differences between the three groups (Table 1). The vital tumor mass was the largest component, accounting for about 50% of the tumor volume in both non-metastatic and lymphogenous-metastatic tumors.

As a next step we ascertained the tumor/necrosis ratio and the tumor/desmoplasia ratio. The tumor/necrosis ratio ranged from 0.5–70 for N0/M0, 0.75–40 for N+ and 0.5–12 for M+ with the median at 5.5 (Table 1). Abundant necrosis was found in 68% of cases with distant metastasis and only in 41% of metastatic-free cases. This difference was just below the statistical significance level (p = 0.073) (Table 2).

The tumor/desmoplasia ratio ranged from 0.43–5.33 in N0/M0, 0.5–5.33 in N+ and 0.5–6 in M+ with the median at 1.5 (Table 1). A prominent desmoplastic stromal reaction was shown in 67% of cases with lymph node metastasis and only in 41% in metastases-free cases. In the binary logistic regression model the nodal positive cases were strongly associated with a low tumor/desmoplasia ratio (p = 0.049) (Table 2).

**Figure 1:**
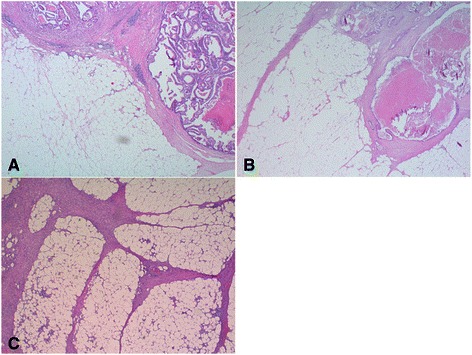
**Desmoplastic pattern in CC.** Pushing **(A)**, finger-like **(B)** and net-like **(C)** pattern of desmoplasia in CC (H.E., x12,5).

Desmoplasia showed three different patterns, pushing, finger-like and net-like (Figure 1). The pushing pattern dominated in lymphogenous-metastatic patients whereas the finger-like pattern was commonest in non-metastatic cases (Table 1). In both groups 46% of the cases expressed the respective phenotype of peritumoral stromal reaction, so that a strong tendency just below statistical significance was observed (p = 0.051) (Table 2). The net-like pattern was seen most frequently in haematogenous-metastatic cases.

#### Blood vessels

**Figure 2:**
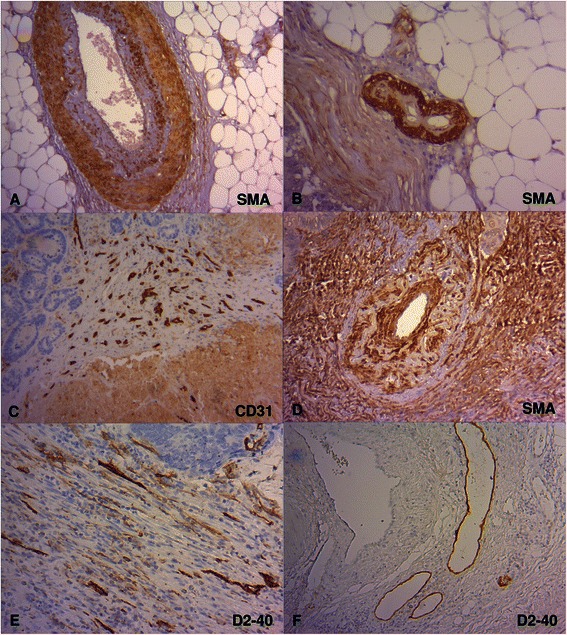
**Morphology of blood and lymphatic vessels in CC. (A)** Large vessels (sm-actin (SMA), x100), **(B)** small vessels (SMA, x200) and **(C)** microvascular vessels (CD31, x40) in CC. **(D)** Altered blood vessels with discontinuously hypoplastic smooth muscle cell layer (SMA, x200). **(E)** Intratumoral compressed lymphatic vessels (D2-40, x200). **(F)** Lymphatic vessels at the tumor periphery with open lumina (D2-40, x100).

To evaluate the histological vascularization pattern of CC we performed immunostaining for the pan-endothelial marker CD31 and smooth muscle marker sm-actin which produced very strong signals in blood vessels of various sizes, that is, large and small vessels as well as microvasculature (Figure 2A-C). The distribution and density of these different sized vessels were analyzed in the tumor center, tumor margin and tumor-free surrounding tissue.

**Figure 3:**
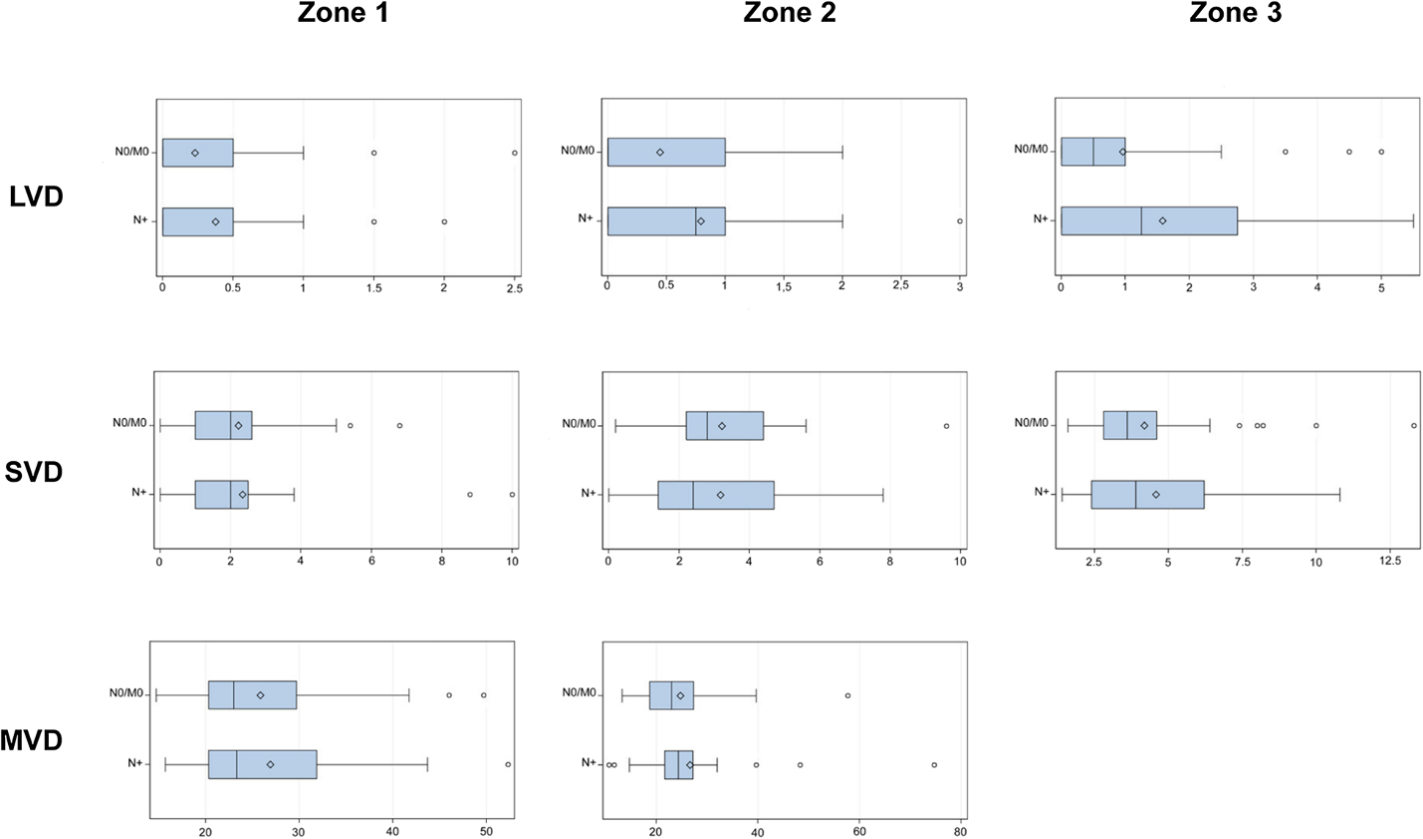
**Vascular densitiy in three different zones in non-metastatic (N0/M0) and lymphogenous metastatic (N+) CC.** Box plot of large vessel density (LVD), small vessel density (SVD) and microvascular vessel density (MVD) in zone 1, zone 2 and zone 3 between non-metastatic (N0/M0) and lymphogenous metastatic (N+) CC. The dark lines denote the median and the open diamond the mean value of each group.

**Figure 4:**
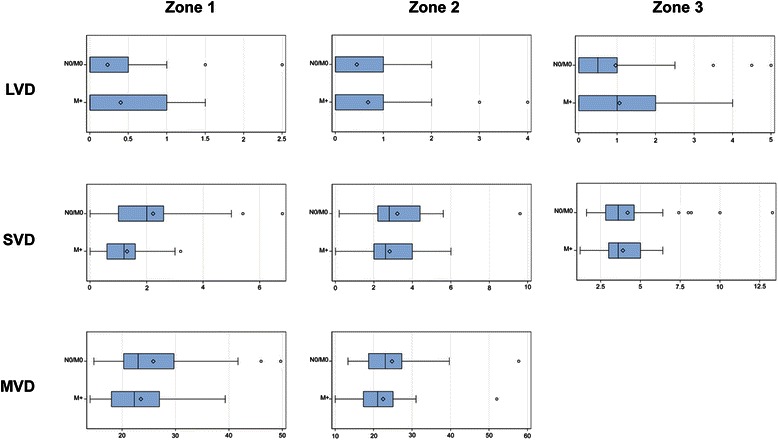
**Vascular densitiy in three different zones in non-metastatic (N0/M0) and haematogenous metastatic (M+) CC.** Box plots of large vessel density (LVD), small vessel density (SVD) and microvascular vessel density (MVD) in zone 1, zone 2 and zone 3 between non-metastatic (N0/M0) and haematogenous metastatic (M+) CC. The dark lines denote the median and the open diamond the mean value of each group.

Large vessels, essential for sufficient blood flow, were seen not only in zone 3 but also intratumorally and along the tumor front (Figures 3 and 4). It should be emphasized that these arteries showed arteriosclerotic changes, characterizing preexistent vessels. In zone 1 and 2 no significant correlations were found between LVD and metastatic status. In zone 3 nodal positive cases were associated with a significantly higher LVD compared to cases without metastasis (p = 0.043) (Table 2). LVD in N0/M0 ranged from 0 – 5.0/25 mm^2^ with the median at 0.5/25 mm^2^ and in N+ from 0 – 5.5/25 mm^2^ with the median at 1,3/25 mm^2^ (Figure 3).

Small vessels, responsible for the maintenance of a densely branched vascular network, were seen at different intensity levels in all three zones (Figures 3 and 4). In zone 1 cases with distant metastasis were associated with a lower SVD compared to N0/M0 (p = 0.013) (Table 2). SVD in N0/M0 ranged from 0 – 6.8/4 mm^2^ with a median value of 2,0/4 mm^2^ and in M+ from 0 – 3.2/4 mm^2^ with a median of 1.2/4 mm^2^ (Figure 4). SVD in zone 2 and 3 revealed no significant correlation with metastatic status.

MVD is known as a histological biomarker reflecting the angiogenetic phenotype of the tumor. In the binary logistic regression model with cut-off at the median no significant correlation regarding metastatic status was verifiable. The distribution of MVD between the three investigated groups was similar in zone 1 as well as in zone 2 (Figures 3 and 4). In zone 1 MVD in N0/M0 ranged from 14.7 – 49.7/mm^2^ (mean 25.8/mm^2^), in N+ from 15.7-52.3 (mean 26.9/mm^2^) and in M+ from 14.0 – 39.3/mm^2^ (mean 23.5/mm^2^). In zone 2 MVD in N0/M0 ranged from 13.3 – 57.7/mm2 (mean 24.8/mm2), in N+ from 10.7-74.7(mean 26.6 /mm^2^) and in M+ from 10.0 – 52.0/mm^2^ (mean 22.4 /mm^2^).

After closer examination of the blood vessel morphology using sm-actin for detailed visualization of the vascular muscle layer a mural alteration was noticed in some of the vessels (Figure 2D). Typically, the tunica media is a layer of continuously concentrically-arranged smooth muscle and elastic fibers. Some blood vessels showed smooth muscle cell hypoplasia with disorganized myogenic fragments and replacement by connective tissue. The distribution of these altered tumor-associated blood vessels within the three zones and among the three groups was strikingly similar (Table 3). In all three tumor compartments in about 33% of the cases this interesting histopathological feature could be demonstrated and was independent of metastatic status.

**Table 3:** **Percentage distribution of altered blood vessels and lymphatic vessels according to their functional status intratumorally (zone 1), along the invasive front (zone 2) and extratumorally (zone 3) in non-metastatic (N0/M0), lymphogenous-metastatic (N+) and haematogenous-metastatic (M+) colon carcinomas**

	N0/M0	N+	M+
**Altered vessels zone 1**			
present	38	33	36
absent	62	67	64
**Altered vessels zone 2**			
present	30	38	32
absent	70	62	68
**Altered vessels zone 3**			
present	35	33	28
absent	65	67	72
**Lymphatic vessels zone 1**			
absent	3	9	4
compressed	78	58	68
mixed	19	33	24
open	0	0	4
**Lymphatic vessels zone 2**			
absent	13	8	16
compressed	38	42	56
mixed	46	42	24
open	3	8	4
**Lymphatic vessels zone 3**			
absent	30	21	24
compressed	11	12	16
mixed	16	17	24
open	43	50	36

Altered vessels were defined as blood vessels showing a discontinuous, hypoplastic smooth muscle cell layer (see Figure 2D).

#### Lymphatic vessels

To detect lymphatic vessels sections were stained with the specific lymphatic endothelial marker D2-40. In almost all investigated cases intratumoral lymphatics were seen (Table 3). Only in 16% and 17% respectively of non-metastatic and lymphogenous metastatic and in 20% of the haematogenous metastatic cases lymphatics were not detectable in tumor-associated regions. Under normal circumstances lymphatic vessels are partially or fully collapsed due to the lack of smooth muscle coverage and low pressure within the lymphatic system. However, for lymphatic vessel invasion open lumina are an important condition. For that reason we focused our attention on the functional state of lymphatic vessels in the three tumor zones. In this context, we recognized three functional lumen patterns, predominantly only compressed, predominantly only open and partly compressed/partly open lumina. Interestingly, whereas most of the intratumoral lymphatics were compressed, an increasing number of open lymphatics along the invasive tumor margin was observed and a clear dominance of lymph vessels with open lumina could be seen in zone 3 (Figure 2E and F). No significant differences could be established among the three metastatic groups (Table 2).

Due to the fact that serial sections were not analyzed a statement concerning the relevance for lymphangiosis and haemangiosis carcinomatosa cannot be made.

### Multivariate analysis

Factors influencing nodal metastasis in the univariate analysis included high tumor budding, high large vessel density in the surrounding tumor-free tissue abundant desmoplasia, non-finger-like desmoplastic pattern and absent lymphocyte-containing intratumoral inflammation. In the multivariate analysis, except LVD, these pathomorphological features were independent risk factors for lymphogenous metastasis (p = 0.023, p = 0.017, p = 0.037, p = 0.012, respectively) (Table 4).

**Table 4:** **Multivariate logistic regression analysis showing associations of relevant histopathological features in non-metastatic (N0/M0) versus lymphogenous-metastatic (N+) and haematogenous-metastatic (M+) CC**

	N0/M0 vs. N+		N0/M0 vs. M+	
	OR (95% CI)	p-value	OR (95% CI)	p-value
**Tumor budding** low vs. high	5.99 (1.28-27.90)	**0.023**	8.70 (1.83-41.42)	**0.007**
**Inflammation zone 1** lymphocytic vs.non-lymphocytic	0.06 (0.01-0.52)	**0.012**	0.20 (0.03-1.38)	NS
**Tumor/Necrosis ratio** (> median, ≤median)			0.46 (0.13-1.62)	NS
**Tumor/Desmoplasia ratio** (> median, ≤median)	0.17 (0.04-0.73)	**0.017**		
**Desmoplastic pattern** finger-like vs. non-finger-like	0.18 (0.03-0.89)	**0.037**		
**Large vessel density zone 3** (> median, ≤median)	2.437 (0.61-9.69)	NS		
**Small vessel density zone 1** (> median, ≤median)			0.33 (0.09-1.23)	NS

OR: odds ratio, CI: confidence interval, NS: no significant.

**Figure 5:**
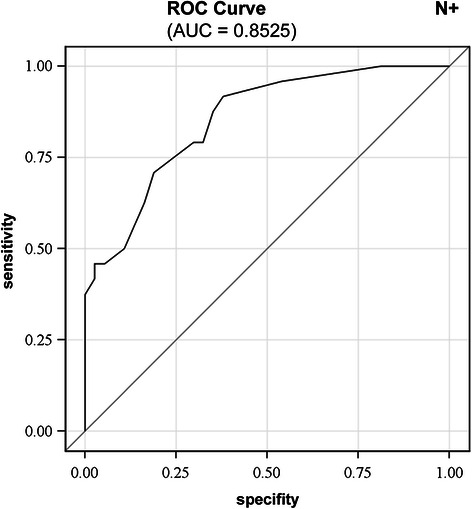
**Receiver-operator characteristic (ROC) curve for predicting nodal metastasis in CC.** ROC curve indicating the sensitivity and specificity of the relevant histopathological features (tumor budding, lymphocellular intratumoral inflammation and abundant desmoplasia with non-finger-like pattern) for predicting nodal metastasis in CC.

Taking into account these identified independent factors, their ability to discriminate between non-metastatic and lymphogenous metastatic group was assessed by a ROC curve. The vertical axis represents sensitivity, which is the proportion of predicted metastatic cases among patients with lymph node metastasis, and the horizontal axis represents specifity, which is the proportion of predicted non-metastastic cases among patients who were *de facto* without metastasis. The more concave the curve, the better the prediction ability. The area under the curve (AUC) is a numerical assessment of concavity. An AUC of 0.5 indicates that prediction is based purely on random chance, and an AUC of 1 indicates that the outcome can be perfectly predicted. In our study the AUC for nodal metastasis was 0.853 suggesting good discrimination (Figure 5).

**Figure 6:**
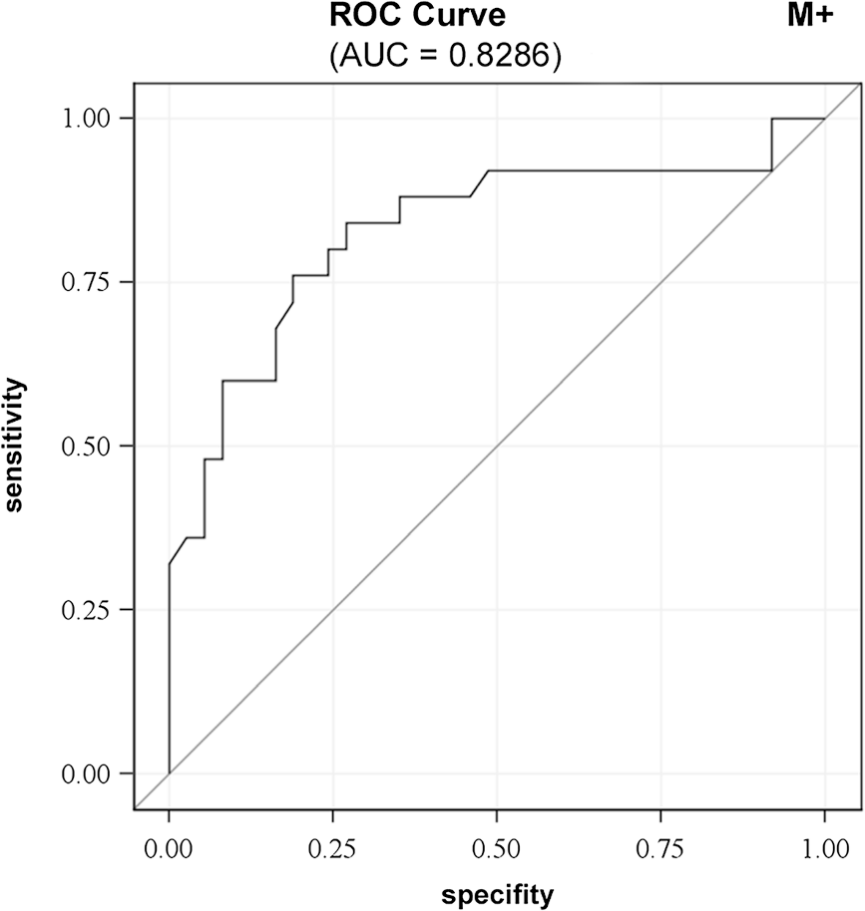
**Receiver-operator characteristic (ROC) curve for predicting distant metastasis in CC.** ROC curve indicating the sensitivity and specificity of tumor budding as solely relevant histopathological feature for predicting distant metastasis in CC.

Factors associated with distant metastasis in the univariate analysis included high tumor budding, low intratumoral small vessel density, absent lymphocyte-containing intratumoral inflammation and abundant necrosis. In the multivariate analysis only tumor budding was proven to be an independent predictor for haematogenous metastasis (p = 0.007) (Table 4). Figure 6 illustrates good discrimination ability (AUC of 0.829).

Notably, in none of the investigated factors could significant differences between lymphogenously and haematogenously metastatic cases be assessed.

## Discussion

This study is one of the first attempts to identify relevant biomarkers in predicting metastasis in CC by detection of structural differences between non-metastatic, lymphogenous-metastatic and haematogenous-metastastic cases in conventional histological sections. From all investigated histological features in tumor tissue of CC high tumor budding, defined as single tumor cells or small tumor cell clusters (≤5 cells) sprouting in more than a third of the total invasion front, emerged as the most convincing predictive factor for both lymph node and distant metastasis. In the literature a strong association between tumor budding and the presence of lymph node metastases is well documented, especially in early submucosa-invasive tumors [19-23]. The significance of tumor budding for distant CC metastasis has received little attention as yet. However, two studies showed that the presence of tumor budding correlates with liver and peritoneal metastases [24,18]. In the present study high tumor budding was the only independent factor in multivariate analysis associated with distant metastasis. The prevailing hypothesis concerning the biological importance of tumor budding is that it represents an epithelial-mesenchymal transition, a biological process characterized by increased migratory capacity and invasiveness [25]. Additionally, it has been hypothesized that budding cells have cancer stem cell character, resulting in enhanced invasive and metastatic properties [26,27]. Despite different scoring systems lacking standardized graduation, tumor budding has proved to be a reliable prognostic marker for CC [28]. In this context, our results underline the fact that this histomorphological feature is an independent predictive factor not only for lymph node but also for distant metastasis of locally advanced CC with good discrimination ability. Tumor budding could be used as a target to discriminate between different aggressive behavior patterns in subserosa-invasive, moderately differentiated CC. In view of these data, a histopathological report should include information on tumor budding, beyond the obligatory TNM-status and grading. The establishment of a consistent, generally accepted, simple applicable scoring system is necessary.

In our study absence of intratumoral lymphocytic inflammation was another histological characteristic associated with nodal and distant metastases. In the multivariate analysis this observation was an independent predictor for lymph node metastasis, but not for distant metastasis. In nearly all non-metastasizing cases lymphocyte infiltration, whether pure or combined with neutrophils, was found within the tumor. These results indicate a predominant role for a tumor-associated adaptive immunity within the tumor itself in the prevention of tumor progression in CC. The protective role of lymphocytes in CC was discussed by a number of study groups. In an overview article Roxburgh and McMillan [29] presented the published data on the relationship between tumor inflammatory cell infiltration and patient survival over a 40 year period. Collectively, the presence of tumor-infiltrating lymphocytes indicates a favorable prognosis. In this context, the comparison between tumors of the same local anatomical extent in the present study suggests a tumor-infiltrating lymphocyte-dependent decreased metastatic potential. In accordance with our results another study demonstrated, that CC metastases in lymph nodes and in the liver are detected when the immune infiltrate in the primary tumor tends to be scarce [30]. In rectal cancer an increased Dukes stage was associated with less peritumoral lymphocyte infiltrate [31]. Interestingly, in virtually all investigated cases independent of the metastatic status a lymphocyte-containing inflammatory response was found in the tumor margin. In this context, Zlobec et al. [32] reported that lymphocytic inflammation at the invasion front does not appear to be an independent prognostic factor in CC. However, gene expression profiling and immunohistochemistry with evaluation of immune cell subtypes demonstrated that type, density and location of immune cells within the tumor samples seem to be a better predictor of patient survival than the histopathological methods currently used to stage CC [33]. The present study has focused only on the inflammatory cell types detected by conventional H.E. staining and their distribution intratumorally and at the invasive front. Furthermore, it assessed neither their density nor the immunhistochemically determined detailed composition and functional state of immune cell subsets. Nonetheless, our preliminary findings indicate that the daily routine H.E. stained tissue sections are a convenient and inexpensive way to study intratumoral inflammation and to gain valuable information on probable tumor metastatic behavior.

In CC, studies of desmoplasia give conflicting results. Several studies suggest that a peritumoral stromal response is an adverse prognostic factor [34-36]. By contrast, Coporale et al. [37] favor the view that desmoplasia is a protective factor for patient survival. In 2D and 3D co-culture systems the cell-cell contact between fibroblast and CC cells evoked an increase of extracellular matrix density and led to inhibited tumor migration and invasion [38]. In our study, abundant desmoplasia (tumor/desmoplasia ratio ≤ 1.5) was an independent predicting factor for lymph node metastasis. Besides differences in the quantity of desmoplasia, we also observed distinct expansion patterns without knowledge of their biological significance. While positive nodal cases showed mostly a pushing desmoplastic stroma pattern, non-metastatic cases exhibited a finger-like pattern. This characteristic spatial extent of desmoplasia was also an independent predictor for nodal status in the multivariate analysis.

Tumor necrosis is a common histological feature in colon cancer, but its biological significance is unclear. So far, only two studies describe the correlation between the extent of histologic tumor necrosis and the TNM-stage. Both research groups reported that extent of necrosis was associated significantly with lymph node metastasis [13,14]. In our study abundant necrosis (tumor/necrosis ratio ≤ 5.5) was seen tendentially more often in cases with distant metastasis. The amount of necrosis in metastatic-free and nodally positive cases was approximately equal. Further investigations are required to determine the role of tumor necrosis in CC metastasis.

The tumor tissue of CC was composed of three basic histological components, namely the cancer cells themselves, the (desmoplastic) interstitium and the necrotic areas. The viable carcinomatous glands were the largest component accounting about 50% of the tumor volume in both non-metastatic and lymphogenous-metastatic tumors. It is clear that the establishment of a functionally integrated vascular system is essential for tumor growth and metastasis. In the present study the density of large and small blood vessels as well as the microvasculature in three strategically significant regions, namely intratumoral, invasive margin and adjacent host tissue, were chosen as a quantitative parameter to describe the vascular network. The large vessels were not only seen in the surrounding subserosal adipose tissue but also within the tumor and along the invasion front. The detection of arteriosclerotic change gave evidence of preexisting vessels. This observation is remarkable, for large vessels are usually not found within the muscle wall and the parts closer to the intestinal lumen. We suggest that the traction effect of the desmoplasia could play an important role by adducting these vessels into the tumor center and front.

Since macrovasculature is responsible for abundant blood transport, this vascular concentration is essential for adjacent nutrition supply. In our study lymphogenous-metastatic CC showed significantly more often a relatively high density of large vessels in the tumor-free peritumoral region. Haematogenous-metastatic CC presented significantly more often a relatively low density of small vessels in the intratumoral region. Since these vessels form a network that regulates local blood perfusion, this fact could explain the abundant necrotic areas in these cases. To our knowledge these observations have not been described in conventional histological sections in detail before. Additionally, in about one third of the CC and independent of the metastatic status we found altered blood vessels with discontinuously hypoplastic muscle wall layers, intra- as well as extratumorally. We hypothesize that these vessels could reflect immature tumor vascular entities, which enable tumors to adapt more easily to extremely harsh conditions because of their leaky architecture with higher fluid and solute exchange ability. In addition, these immature vessels could enhance tumor cell entry into the circulation and hence distant metastasis. In another study immature microvessels without pericyte coverage were described in correlation with haematogenous metastasis of CC [39]. Microvascular density (MVD) is considered to be the morphological gold standard to assess the neovasculature in human tumors [40]. A meta-analysis found a statistically significant inverse relationship between MVD and survival in CC [41]. In our study MVD measurement in central tumor portions and at the surrounding part of the tumor was without significant differences between non-metastatic and metastatic CC. Our results indicate that MVD is not sufficient to predict metastasis in locally advanced CC. In this tumor status, the evaluation of expression of angiogenic factors in tumor specimens might provide an alternative to MVD in assessing tumor angiogenic activity with more functional information.

In almost all CC intratumoral lymphatics were detectable but were mostly collapsed and showed compressed lumina. In contrast to this, directly beneath the deep infiltration front in the peritumoral region and mainly in the tumor-free surrounding tissue lymphatic vessels with open lumina predominated. This morphological distribution suggests a prevalent role of peritumoral lymphatics in lymphogenous metastasis, because collapsed intratumoral lymphatics are not suitable for tumor cell dissemination. Collapsed intratumoral lymphatics could reflect a mechanical stress situation induced by the vessel pressure in the desmoplastic tumor tissue. By a compensatory mechanism the resulting elevated lymph pressure could lead to a dilatation of the lymphatics in the tumor periphery. Another research group has made the same observations in CC using color stereoscopic lymphography [42]. We conclude that the tumor margin and the surrounding tissue with open lymphatics are the most favorable site of entry of tumor cells into lymphatics. Notably, no significant differences between non-metastatic and metastatic cases were found concerning this specific lymphovascular histoarchitecture.

## Conclusions

Taken the results together, tumor budding represents an independent predictive biomarker with a good discrimination ability for both lymphogenous and haematogenous metastasis in moderately differentiated, subserosa-invasive CC. Absence of intratumoral lymphocytic inflammation and abundant desmoplasia with non-finger-like pattern could provide additional independent predictive factors for lymph node metastasis. Abundant tumor necrosis with low intratumoral small vessel density is a common feature of distant metastasis. Thus, tumor budding and the local peritumoral situation seem to be able to reflect the metastatic potential. A statement of these histological features in the histopathological report could provide an ancillary tool for the further imaging and therapeutic management after surgery. Since metastatic dissemination could exist before a pathologically and clinically detectable manifestation, these features could also be considered in metastatic-free cases in locally advanced CC.
